# Pink edematous papules and plaques of the trunk and extremities

**DOI:** 10.1016/j.jdcr.2021.05.022

**Published:** 2021-05-26

**Authors:** Shadi Khalil, Brian Hinds, Joyce Y. Cheng, Bonita Kozma

**Affiliations:** Department of Dermatology, University of California, San Diego, California

**Keywords:** borderline lepromatous leprosy, Hansen's disease, host immunity, leprosy, *Mycobacterium leprae*

A 72-year-old man presented to our outpatient dermatology clinic in San Diego, California, complaining of a 3-month progressive asymptomatic rash that began appearing on his left leg (Fig 1) and spread to involve his right leg, trunk, face, and upper extremities (Fig 2). History was notable for remote travel to Mexico and the Philippines. The patient was born in the Philippines and moved to the United States 30 years ago. The review of systems was negative for weight loss, night sweats, cough, fever, shortness of breath, skin pain, or sensory changes to the involved skin. Past medical history was unremarkable. No new medications were started prior to the onset of the rash. Physical examination noted numerous edematous pink papules and plaques of the trunk, face, arms, and legs. Histopathologic examination of a skin biopsy specimen showed extensive granulomatous dermal inflammation (Fig 3).

**Fig 1:**
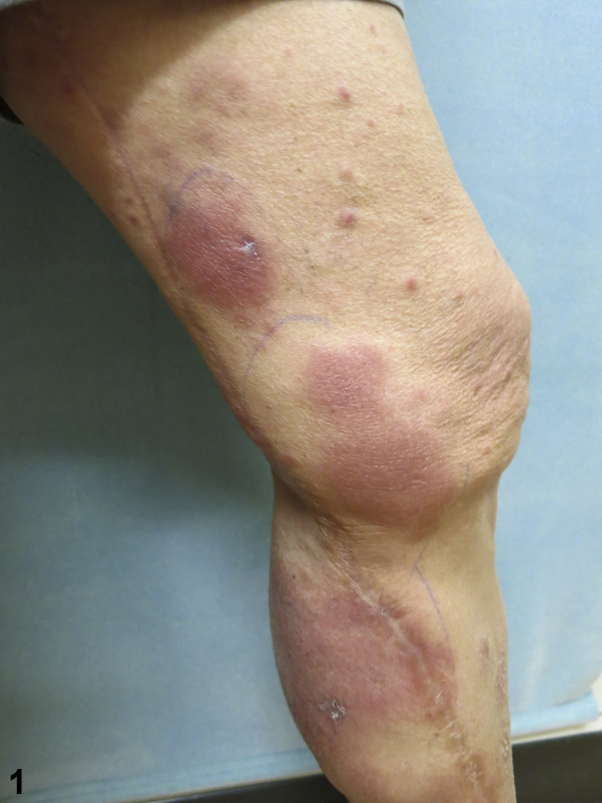

**Fig 2:**
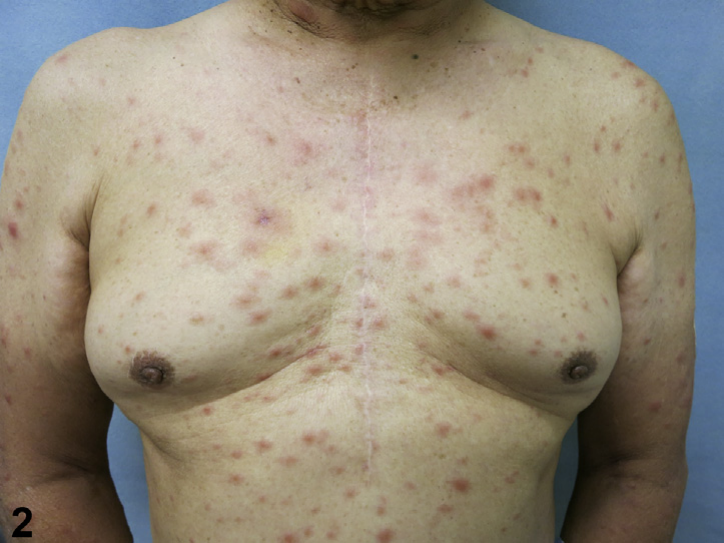

**Fig 3:**
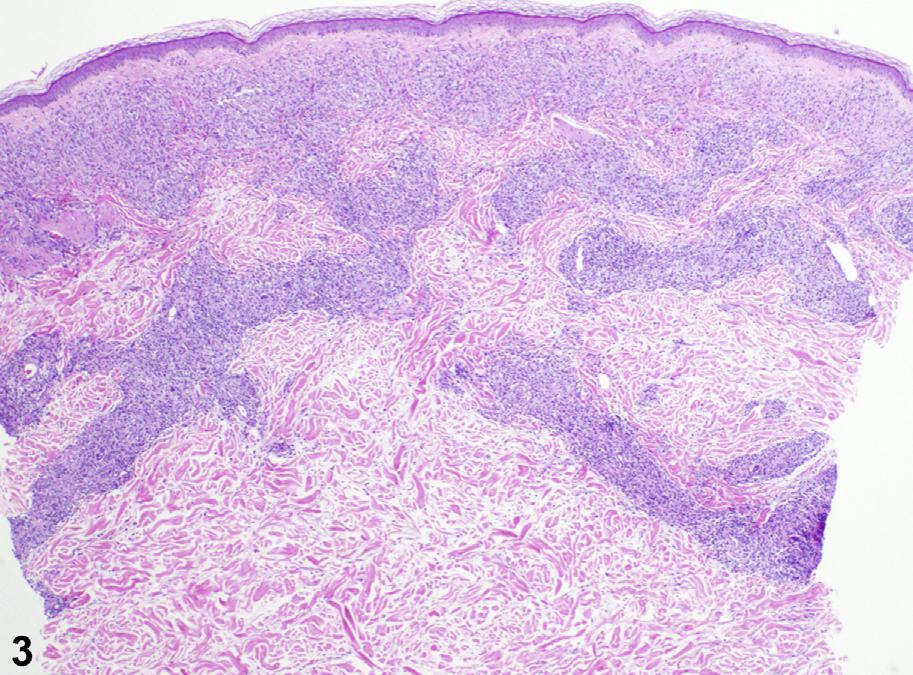

**Question 1: What is the best diagnosis?**

A. Cutaneous tuberculosis
B. Sarcoidosis
C. Hansen's disease
D. Cutaneous lymphoma
E. Disseminated granuloma annulare

**Answers:**

A. Cutaneous tuberculosis – Incorrect. Cutaneous tuberculosis is an appropriate diagnosis to consider but unlikely given the widespread distribution without the red flag clinical symptoms (weight loss, fever, or night sweats). The histology of cutaneous tuberculosis would likely include necrotizing granulomas, which were not observed.
B. Sarcoidosis – Incorrect. Similar to syphilis, sarcoidosis is a great mimicker with potential for various clinical presentations. Histologically, sarcoidosis is less likely to present with a curvilinear histiocytic infiltrate pattern following the neural bundles. “Naked” granulomas would be expected.
C. Hansen's disease – Correct. The clinical presentation, history, and histology demonstrating a histiocytic infiltrate outlining the dermal neurovascular bundles are suggestive of Hansen's disease. The polymerase chain reaction of tissue-derived organisms identified *Mycobacterium leprae*. Hansen's disease, or leprosy, is a spectrum of disease caused by *M leprae* and *Mycobacterium lepromatosis* infections. *M leprae* and *M lepromatosis* are slow-growing intracellular mycobacteria that directly infect macrophages, endothelial cells, and Schwann cells.^1^ Globi of acid-fast bacilli were noted throughout the histiocytic infiltrate by Fite staining. The patient was diagnosed with borderline lepromatous leprosy and initiated on multidrug therapy with rifampin, minocycline, and clofazimine.
D. Cutaneous lymphoma – Incorrect. Although lymphoma may be considered a possible diagnosis, the lack of the red flag clinical symptoms such as weight loss, night sweats, or fevers suggests a nonmalignant process. Histology was not notable for a prominent monomorphic lymphoid population.
E. Disseminated granuloma annulare – Incorrect. Disseminated or generalized granuloma annulare is less likely given the asymptomatic symmetric papules and plaques with absent annular morphology. The histopathology of granuloma annulare would be expected to show palisaded histiocytes and mucin deposition.

**Question 2: Which of the following countries had the highest rate of new infections in 2019?**

A. Brazil
B. India
C. Mexico
D. Philippines
E. The United States

**Answers:**

A. Brazil – Correct. The majority of new worldwide cases occur in a few countries in which the infection is less controlled. Brazil, India, and Indonesia together account for 80% of new worldwide infections. In 2019, the incidence of new cases was the highest in Brazil, which reported over 27,863 cases in a population of 211 million (13 per 100,000 people).^2^
B. India – Incorrect. India had 114,451 new cases in 2019, the highest than any other nation. With a population of 1.3 billion, the rate of new infections (9 per 100,000 people) is slightly below than that of Brazil.
C. Mexico – Incorrect. In 2019, Mexico reported 182 new cases in a population of 126 million (rate of 1.4 per 100,000 people). Mexico is a common country of origin in patients with new diagnoses in Southern California.^3^
D. Philippines – Incorrect. The Philippines is one of the 23 countries in the World Health Organization's global priority list that represent over 95% of the new worldwide Hansen's disease infections. With 2122 new infections in 2019 in a population of 108 million (rate of 2 per 100,000 people), it had considerably fewer new infections than Brazil, Indonesia, or India. Of note, the patient in this case did describe a history of remote travel to the Philippines, where he may have been initially infected. Incubation can take as long as 10 years from the initial exposure to the clinical presentation.^3^
E. The United States – Incorrect. The United States sees approximately 200 cases annually, mostly in patients with a history of travel to endemic areas.

**Question 3: Which of the following is a risk factor for developing Hansen's disease?**

A. Adult age
B. Exposure to rats
C. Genetic susceptibility
D. Skin-to-skin contact with an infected patient
E. Exposure to a patient with treated tuberculoid leprosy

**Answers:**

A. Adult age – Incorrect. Children and elderly patients are both at higher risk than adults for developing Hansen's disease following the exposure.
B. Exposure to rats – Incorrect. Rats are not hosts to *M leprae*. Armadillos, present in the southern United States and Brazil, are a natural reservoir for *M leprae*. Zoonotic infection following armadillo exposure (including the consumption of armadillo meat) has been documented.^1^^,^^4^
C. Genetic susceptibility – Correct. Ninety-five percent of people have natural immunity and are not infected following the exposure.^1^^,^^2^ Genetic susceptibility is a major risk factor and is determined by risk alleles in the genes that are essential for cell-mediated immunity, including IFNG, TLR1, SOD2, and interleukin 10.^5^ In the minority of patients in whom the Hansen's disease develop following the exposure, host immunity further defines the type of leprosy. Tuberculoid leprosy occurs with sufficient cell-mediated immunity and suppression of bacterial proliferation, whereas lepromatous leprosy represents humoral-skewed immunity with increased bacterial proliferation and burden. The patient in this case presented with a widespread disease that was insufficient for the diagnosis of lepromatous leprosy and was thus diagnosed with borderline lepromatous leprosy.
D. Skin-to-skin contact with an infected patient – Incorrect. Infection by *M leprae* and *M lepromatosis* occurs through infectious respiratory droplets and requires a prolonged close contact. Nasal mucosa demonstrates significantly higher levels of bacteria than either blood or skin.^6^
E. Exposure to a patient with treated tuberculoid leprosy – Incorrect. Patients with tuberculoid leprosy have fewer bacterial organisms in their nasal secretions, skin, and blood.^4^ Although all forms of Hansen's disease are contagious, contact with patients with lepromatous leprosy confers a higher risk of infection than the other forms of leprosy.^1^^,^^4^ Initiating treatment for both subtypes of leprosy rapidly decreases the transmission risk.

## Conflicts of interest

None disclosed.
